# Ipragliflozin-induced adipose expansion inhibits cuff-induced vascular remodeling in mice

**DOI:** 10.1186/s12933-019-0886-1

**Published:** 2019-06-24

**Authors:** Kentaro Mori, Kyoichiro Tsuchiya, Suguru Nakamura, Yasutaka Miyachi, Kumiko Shiba, Yoshihiro Ogawa, Kenichiro Kitamura

**Affiliations:** 1 Third Department of Internal Medicine, Interdisciplinary Graduate School of Medicine and Engineering, University of Yamanashi, 1110 Shimokato, Chuo, Yamanashi 4093898 Japan; 2 Department of Molecular Endocrinology and Metabolism, Graduate School of Medical and Dental Sciences, Tokyo Medical and Dental University, Tokyo, Japan; 3 Department of Medicine and Bioregulatory Science, Graduate School of Medical Sciences, Kyushu University, Fukuoka, Japan; 4 Department of Molecular and Cellular Metabolism, Graduate School of Medical and Dental Sciences, Tokyo Medical and Dental University, Tokyo, Japan; 5 Japan Agency for Medical Research and Development, CREST, Tokyo, Japan

**Keywords:** Vascular remodeling, Perivascular adipose tissue, Healthy adipose expansion, Cuff injury

## Abstract

**Background:**

Perivascular adipose tissue (PVAT) plays a critical role in the pathogenesis of cardiovascular disease. It is unclear whether inhibition of sodium glucose cotransporter 2 (SGLT2) in subjects with type 2 diabetes (T2DM) could affect PVAT characters, and whether the SGLT2 inhibitors-induced changes of adipose tissue, especially the alternation of adipose tissue-derived secretory factors, affect vascular pathophysiology.

**Methods:**

Western-type diet (WD) fed wild-type mice were treated with or without an SGLT2 inhibitor ipragliflozin (Ipra) for 10 weeks. WEHI 274.1 and primary vascular smooth muscle cells were incubated with conditioned media (CM) of epididymal adipose tissue (Epi) or abdominal PVAT of Ipra- or vehicle-treated mice fed a WD. Epi of Ipra- or vehicle-treated mice fed a WD was implanted onto cuff-placed femoral arteries of apoE-deficient mice.

**Results:**

Ipra increased adipocyte size associated with decreased expression of pro-inflammatory and fibrosis-related genes in abdominal PVAT of WD-fed mice. Ipra also suppressed WD-induced macrophages accumulation, fibrosis, and adipocyte death in abdominal PVAT. In CM of abdominal PVAT from Ipra-treated mice, concentration of leptin was significantly lower than that from vehicle-treated mice. In vitro, migration of WEHI 274.1 and primary vascular smooth muscle cells were more enhanced by CM of Epi or abdominal PVAT from vehicle-treated mice than that from Ipra-treated mice. Perivascular implantation of Epi from Ipra-treated mice to apolipoprotein E-deficient mice attenuated cuff-induced neointimal hyperplasia and vascular remodeling compared to that from vehicle-treated mice.

**Conclusions:**

The Ipra-induced changes of abdominal PVAT will lead to a better understanding of unveiled mechanisms by which SGLT2 inhibitors prevent cardiovascular complications in T2DM, and the development of new therapeutic strategies targeting PVAT.

**Electronic supplementary material:**

The online version of this article (10.1186/s12933-019-0886-1) contains supplementary material, which is available to authorized users.

## Background

Sodium glucose cotransporter 2 (SGLT2) inhibitors are a class of oral hypoglycemic agents that work by decreasing glucose reabsorption in the renal proximal tubules to promote urinary glucose excretion. The net effect of increased renal glucose excretion has dual effects of insulin-independent glycemic control and caloric loss, thereby leading to improve insulin sensitivity. Accumulating evidence suggests that SGLT2 inhibitors provide multiple benefits to reduce major cardiovascular adverse events including myocardial infarction, stroke, and heart failure in patients with type 2 diabetes (T2DM) [1, 2]. Recent clinical [3, 4] and experimental [5] evidence have further confirmed that SGLT2 inhibitors have benefits on atherosclerotic cardiovascular events. Although both hemodynamic and metabolic explanations have been proposed, the mechanisms underlying the protective effects of SGLT2 inhibitors on cardiovascular complications among T2DM still remain to be explored.

Perivascular adipose tissue (PVAT) surrounds vasculature and has been suggested to play an important role in the pathogenesis of cardiovascular disease [6]. PVAT not only stores triglycerides and functions as structural support for vessels, but also secretes a wide variety of biologically active molecules to control vascular function as well as remodeling. In obesity or T2DM, PVAT dominantly secrete pro-inflammatory and pro-atherogenic cytokines, which could cause local endothelial dysfunction, thus contributing to the progression of systemic and local vascular diseases. For instance, leptin, visfatin, and monocyte chemoattractant protein-1 (MCP-1), which modulate vascular tone, macrophages recruitment and activation, vascular smooth muscle cells (VSMCs) migration and proliferation [7], are secreted from PVAT to potentially promote neointimal hyperplasia and formation in adjacent blood vessel by the paracrine fashion [8]. Among antidiabetic agents, alogliptin reported to improve endothelial function by promoting autophagy in perivascular adipose tissue of obese mice through a glucagon-like peptide-1-dependent mechanism [9]. As human evidence, secreted frizzled-related protein 4 derived from epicardial adipose tissue, which can be recognized as PVAT of coronary arteries, is reported to increase in patients with coronary artery disease [10]. It therefore suggests that PVAT has been considered as a potential therapeutic target for treatment of atherosclerosis associated with obesity and/or diabetes [11].

Others and we recently reported that the SGLT2 inhibitors promotes fat accumulation in epididymal adipose tissue (Epi) of diet-induced obese mice without deteriorating adipose inflammation and/or fibrosis, which may be referred to as “healthy adipose expansion” [12–16]. However, it remains unknown whether SGLT2 inhibitors also alter characters of PVAT to the “healthy adipose expansion”, and if so, whether the SGLT2 inhibitors-induced changes of adipose tissue, especially the alternation of adipose tissue-derived secretory factors, affect vascular pathophysiology.

Here we demonstrated that treatment with Ipra could increase adipocyte size with decreased expression of pro-inflammatory and pro-fibrotic genes in abdominal PVAT of Western-type diet (WD)-fed wild-type (WT) mice. Ipra also suppressed WD-induced macrophages accumulation and adipocyte death in abdominal PVAT, and perivascular implantation of Epi from Ipra-treated mice to apoE-deficient mice attenuated cuff-induced neointimal hyperplasia and vascular remodeling. This will lead to a better understanding of unveiled mechanisms by which SGLT2 inhibitors prevent cardiovascular complications in T2DM, and the development of new therapeutic strategies focusing on PVAT.

## Materials and methods

### Animals and experimental protocol

Male C57BL/6 J WT mice were purchased from CLEA Japan, Inc. (Tokyo, Japan) and Charles River Laboratories Japan, Inc. (Kanagawa, Japan). Male apolipoprotein-E (ApoE)-knockout mice were purchased from Charles River Laboratories Japan, Inc. The animals were allowed free access to water and a standard diet (SD, CE-2; 343 kcal/100 g, 12.6% energy as fat; CLEA Japan, Inc.). Ipragliflozin (Ipra: provided by Astellas Pharma Inc., Tokyo, Japan) was dissolved in dimethyl sulfoxide (DMSO; Nacalai Tesque, Inc., Kyoto, Japan) at 0.04% (v/v) and added into the drinking water. In a Western type diet (WD) feeding experiments, 8-week-old WT mice were fed a WD (D12079B, 41% fat, 43% carbohydrate, and 17% protein content on an energy basis, Research Diets Inc., New Brunswick, NJ, USA) for 8 weeks, and thereafter a WD with the vehicle or Ipra for 10 weeks. Age matched control male WT mice were fed a SD throughout the experiment period. Body weight and blood glucose were measured every week. Thoracic PVAT from distal end of the aortic arch to the diaphragm, and abdominal PVAT from diaphragm to bifurcation were harvested. Upper and lower half of the PVATs were subjected to histological and mRNA/protein analyses, respectively. Concentration of Ipra in the drinking water was changed every week based on daily water consumption and body weight to adjust 10 mg/kg/day. At the end of the experiment, the animals were sacrificed under intraperitoneal pentobarbital anesthesia (30 mg/kg) after 16 h of fasting.

### Biochemical assays

Blood glucose was measured using a glucometer (OneTouch Verio^®^ IQ; LifeScan Japan Co., Ltd., Tokyo, Japan). Serum total cholesterol, non-esterified fatty acid, and triglyceride (TG) concentrations were determined with Wako Cholesterol E, NEFA C-Test Wako, and TG E-Test Wako (Wako Pure Chemical Industries, Ltd., Osaka, Japan), respectively. Insulin (Morinaga Institute of Biological Science, Inc., Kanagawa, Japan), leptin (FUJIFILM Wako Shibayagi Co., Gunma, Japan), high mobility group box 1 (HMGB1) (FUJIFILM Wako Shibayagi Co.), and fatty acid binding protein 4 (FABP4) (MyBioSource, Inc., CA, USA) concentrations were measured with an enzyme-linked immune sorbent assay kits.

### Histological analysis

The aorta and PVAT were fixed with 4% paraformaldehyde and embedded in paraffin and prepared as slides (4-µm-thick sections). PVAT sections were stained with hematoxylin and eosin (HE). For the measurement of adipocyte cell size, > 250 cells were counted per each section using an image analysis software (NIH Image J software). Macrophages in the PVAT were immunohistochemically detected using a rat monoclonal F4/80 antibody (MCA497GA; Abd Serotec, Kidlington, UK). Neointima formation in cuff-injured femoral arteries were stained as immunohistochemically detected using a α-smooth muscle actin (α-SMA) antibody (ab5694, Abcam, Cambridge, UK). Elastica-van Gieson and Masson trichrome staining were also performed in femoral arteries. Binding of primary antibody was visualized using DAB + chromogen (Dako, Glostrup, Denmark). PVAT was stained using Sirius red for the assessment of fibrosis; positive areas were measured using NIH Image J software. TUNEL staining was performed using the ApopTag Plus Peroxidase In Situ Apoptosis Detection Kit (Millipore, Billerica, MA, USA) according to the manufacturer’s instruction. TUNEL-positive cells were counted in the whole area of the section. All microscopic images were acquired using the Keyence BZ-9000 microscope.

### Quantitative RT-PCR

Total RNA of the PVAT was isolated using Sepasol reagent (Nacalai Tesque, Inc.). RNA was reverse transcribed with Random Primer (Thermo Fisher Scientific Inc., Waltham, MA, USA) and ReverTra Ace (Toyobo Co., Ltd., Osaka, Japan). Quantitative RT-PCR was performed using StepOnePlus Real-time PCR System with Fast SYBR Green Master Mix Reagent (Thermo Fisher Scientific Inc.). Primers are listed in S Table. Data were normalized to the 36b4 levels, and analyzed by the comparative CT method.

### Western blotting

PVAT was homogenized in a lysis buffer (2% SDS, 4 M Urea, 1 mM EDTA, 150 mM NaCl, 50 mM Tris pH 8.0). Immunoblotting was performed with a phospho (Ser473)-Akt (9271, Cell Signaling Technology, Danvers, MA, USA), a total Akt antibody (9272, Cell Signaling Technology), HMGB1 (6893, Cell Signaling Technology), β-actin (4970, Cell Signaling Technology). Immunoblots were detected and analyzed with ECL Prime Western Blotting Detection Reagent and ImageQuant LAS 4000 mini (GE Healthcare, Little Chalfont, UK).

### Conditioned media collection from adipose tissue

Experimental mice (n = 2 in each group) were sacrificed by intraperitoneal pentobarbital anesthesia. The epididymal adipose tissue (Epi) or abdominal PVAT from diaphragm to bifurcation were dissected in DMEM with 1% penicillin/streptomycin (P/S), 0.25% BSA. Tissue pieces from each mice were weighed as 100 mg (Epi) or 20 mg (abdominal PVAT), then minced 20 times, and incubated with 1 ml of DMEM with 1% P/S, 0.25% BSA in 24-well culture plates (Costar, Corning, NY, USA) at 37C for 5 h.

### Chemotaxis assays

The effects of CM of Epi on monocytes recruitment were determined using Boyden chambers (Cell Biolabs, CytoSelect™ 24-Well Cell Migration Assay, 5 μm). Murine monocytes (WEHI 274.1 cells, purchased from ATCC) were initially cultured in DMEM with 10% FBS and 1% P/S, and split twice a week to obtain a sufficient cell number. For experiments, WEHI 274.1 cells were plated into the upper chamber at 100,000 cells per 100 μl DMEM with 1% P/S, while CM diluted twice by DMEM was filled into the lower one with or without anti-MCP-1 blocking antibody (R&D Systems, Inc, MN, USA). After 12 h, the number of migrated cells was counted on dissected membranes using Nikon Eclipse Ti microscope.

### Scratch test

Vascular smooth muscle cells were isolated and cultured from 4-week-old WT male as previously described [17]. Primary VSMCs were plated in 12-well plates at a concentration of 500,000 cells per 2 ml of DMEM with 20% FBS and 1% P/S. The media was changed to DMEM with 0.25% BSA when an adherent monolayer was obtained. Scratch was created by a 100 μl pipette tip in the monolayer and the cells were washed 3 times with DMEM with 0.25% BSA. The cells were then stimulated with rat recombinant platelet-derived growth factor (PDGF)-BB (R&D Systems) in CM diluted twice by DMEM with or without LY294002 (Cell Signaling Technology). After 24 h, images of the scratch wounds were taken and measured by Image-J software.

### Perivascular implantation of adipose tissue and cuff injury

Surgery was carried out on 8-week-old male mice as described previously [18] with some modifications. In brief, the femoral artery was isolated from surrounding tissues under anesthesia, and then a polyethylene tube (PE-20; BD) was loosely placed around the artery. Fifty mg of Epi was taken from WD-fed or WD/Ipra-fed mouse, followed by placed onto the artery after cuff placement. The mice were sacrificed 4 weeks after surgery, and the implanted Epi and arteries were obtained for analyses.

### Statistical analysis

For normally distributed values, data were expressed as mean ± standard error of the mean (SEM), and were compared using student’s t test, or analysis of variance (ANOVA) with post hoc testing. For non-Gaussian distributed values, data were expressed as box-and-whisker plots with median values and 10, 25, 75, and 90‰. Nonparametric statistical analysis was performed with the Mann–Whitney or Kruskal–Wallis test with Dunn post hoc test. *p* < 0.05 was considered to be statistically significant. Statistical analysis was performed using Prism 7 (GraphPad software, Inc., CA, USA).

## Results

### Ipra increases adipocyte size with enhanced insulin signaling in abdominal PVAT of WD-fed mice

**Fig. 1:**
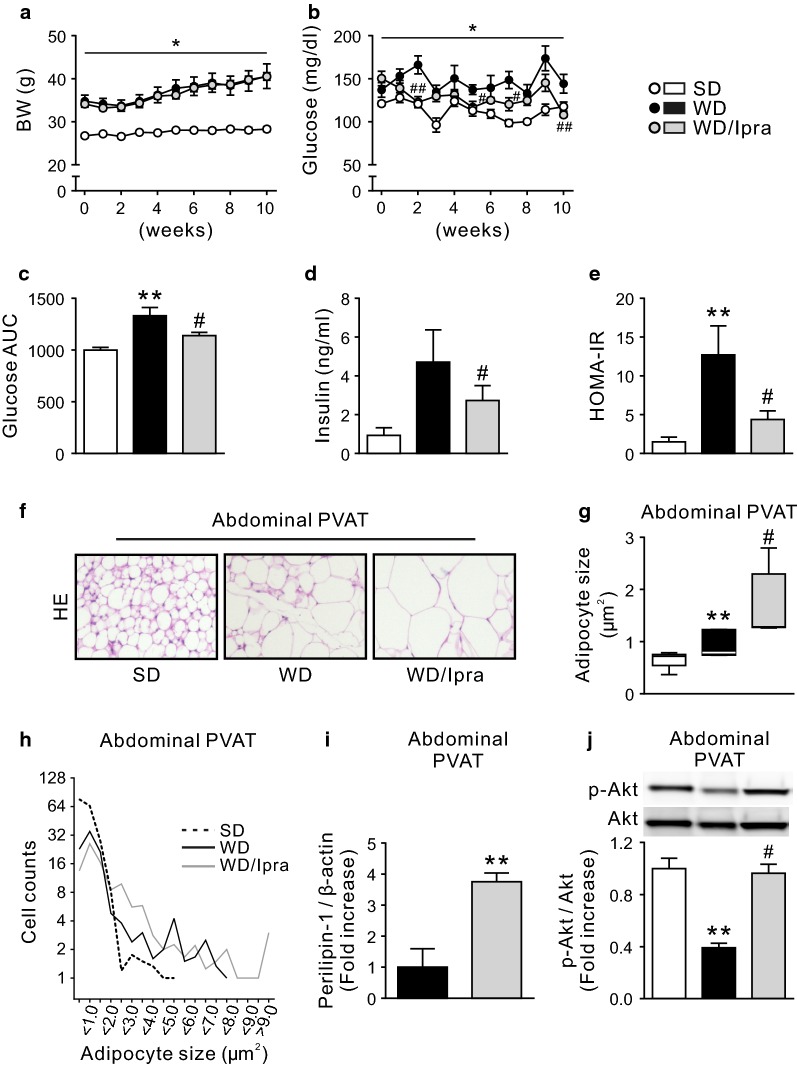
Ipra increases adipocyte size with enhanced insulin signaling in abdominal PVAT of WD-fed mice. The changes in **a** body weight and **b** blood glucose, and **c** area under curve (AUC) of blood glucose in SD- or WD-fed WT mice during Ipra treatment for 10 weeks. **d** Plasma insulin concentration and **e** HOMA-IR after 10 weeks of Ipra treatment. **f** Hematoxylin and eosin (HE) staining, and **g** quantification and **h** histogram of adipocyte size in abdominal PVAT. Quantitative data of **i** perilipin-1 and **j** phosphorylated Akt (p-Akt) in abdominal PVAT. *WT* wild-type, *SD* standard diet, *WD* Western-type diet, *Ipra* ipragliflozin, *PVAT* perivascular adipose tissue. Original magnification, ×200. *p < 0.05, **p < 0.01 vs SD. ^#^*p* < 0.05, ^##^*p* < 0.01 vs WD. n = 6–8

First we put WT mice on WD for 10 weeks with or without Ipra treatment. Ipra did not affect the changes of body weight during the WD feeding as previously shown in mice fed a HFD [19] (Fig. 1a). In contrast, Ipra treatment significantly increased Epi weight to body weight ratio (Additional file 1: Fig. S1a). Ipra significantly attenuated WD-induced hyperglycemia (Fig. 1b, c) and insulin resistance with a trend of decrease of serum insulin concentration (Fig. 1d, e). Ipra did not change serum lipid profile of WD-fed mice (Additional file 1: Fig. S1b). Histological analysis revealed that Ipra treatment increased adipocyte size in abdominal PVAT of WD-fed mice (Fig. 1f–h), accompanied with increased expression of lipid storage marker perilipin-1 (Fig. 1i). Ipra did not affect adipocyte size in thoracic PVAT (Additional file 1: Fig. S1c). Whereas phosphorylation of Akt (Ser473) in abdominal PVAT of WD-fed mice was significantly suppressed compared to SD-fed mice, its suppression was not observed in Ipra-treated mice (Fig. 1j).

### Ipra attenuates inflammation, fibrosis, and adipocyte death in abdominal PVAT of WD-fed mice

**Fig. 2:**
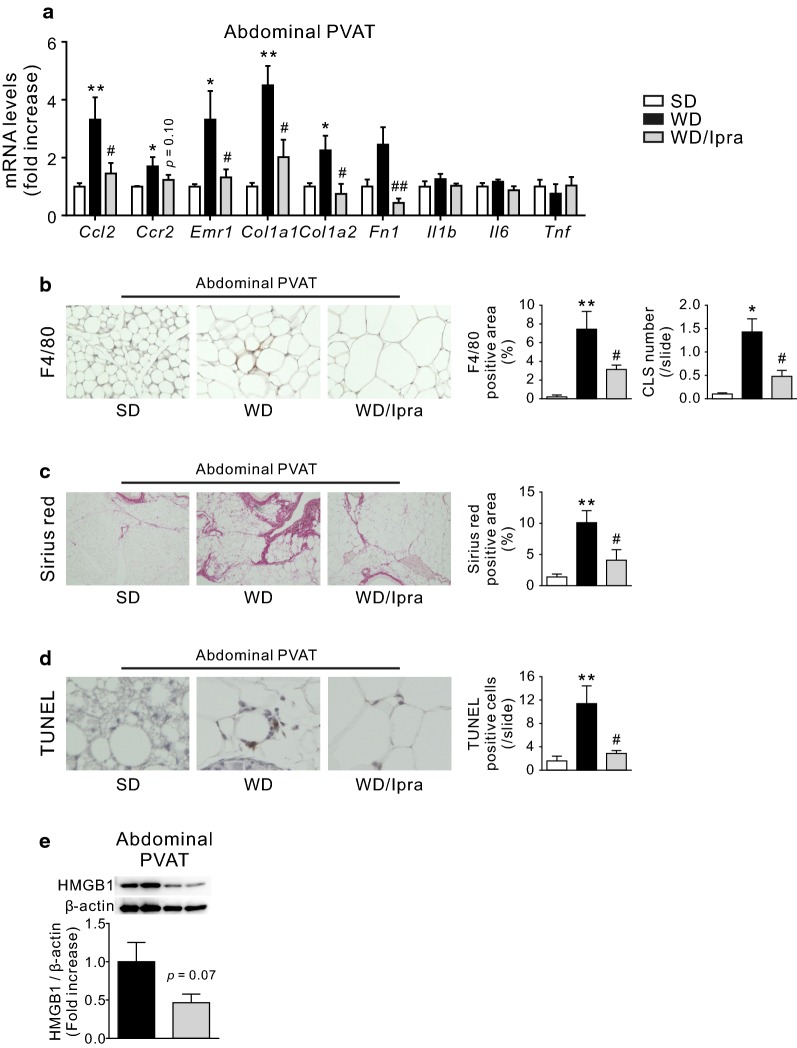
Ipra attenuates inflammation, fibrosis, and cell death in abdominal PVAT of WD-fed mice. **a** Expression levels of inflammation- and fibrosis-related genes in abdominal PVAT of SD- or WD-fed WT mice after 10 weeks of Ipra treatment. **b** Representative pictures of F4/80 immunostaining and quantitative data, and the number of crown-like structure (CLS) in abdominal PVAT. **c** Representative pictures of Sirius red staining and quantitative data in abdominal PVAT. **d** Representative pictures of TUNEL staining and quantitative data in abdominal PVAT. Quantitative data of HMGB1 protein in **e** abdominal PVAT. *Epi* epididymal adipose tisse. Original magnification, ×200 (F4/80), ×40 (Sirius red). *p < 0.05, **p < 0.01 vs SD. ^#^*p* < 0.05, ^##^*p* < 0.01 vs WD. n = 6–8

In abdominal PVAT, inflammation (*Ccl2*, *Ccr2*, and *Emr1*)- and fibrosis (*Col1a1*, *Col1a2*, and *Fn1*)-related genes were upregulated in WD-fed mice compared to SD-fed mice, which were significantly or tended to be inhibited by Ipra treatment (Fig. 2a). Expression of *Il1b*, *Il6*, and *Tnf* were unaffected by WD feeding and Ipra treatment (Fig. 2a). Expression of *Ccl2*, *Ccr2*, and *Emr1* in thoracic PVAT was not changed by Ipra treatment (Additional file 1: Fig. S1d). Accordingly, immunostaining for a macrophage marker F4/80 revealed that Ipra treatment effectively suppressed macrophage infiltration and crown-like structure (CLS) formation in abdominal PVAT of WD-fed mice (Fig. 2b). Fibrosis in abdominal PVAT assessed by Sirius red staining, which is reported to tightly associated with tissue inflammation characterized by macrophage infiltration [20], was also significantly reduced in Ipra-treated mice (Fig. 2c).

It has been reported that the number of macrophages in obese adipose tissue positively correlates adipocyte apoptosis [21]; in consistent with reduced CLS in abdominal PVAT of Ipra-treated mice, Ipra significantly decreased the number of TUNEL-positive cells in WD-fed mice as compared to vehicle-treated mice (Fig. 2d). Dying adipocytes in obese adipose tissue may contribute to the recruitment of immune cells or release of intracellular molecules of adipocytes known as damage-associated molecular patterns [22]. A nuclear protein, high mobility group box 1 (HMGB1), is one of the damage-associated molecular patterns and released from dying adipocytes to potentially promote atherosclerosis and vascular remodeling [23, 24]. As expected, Ipra tended to reduce protein expression of HMGB1 in abdominal PVAT (Fig. 2e), and suppressed HMGB1 release from isolated Epi of WD-fed mice into CM (data not shown).

### Ipra-induced changes of secretory factors from Epi or abdominal PVAT inhibit monocytes and VSMCs migration in vitro

**Fig. 3:**
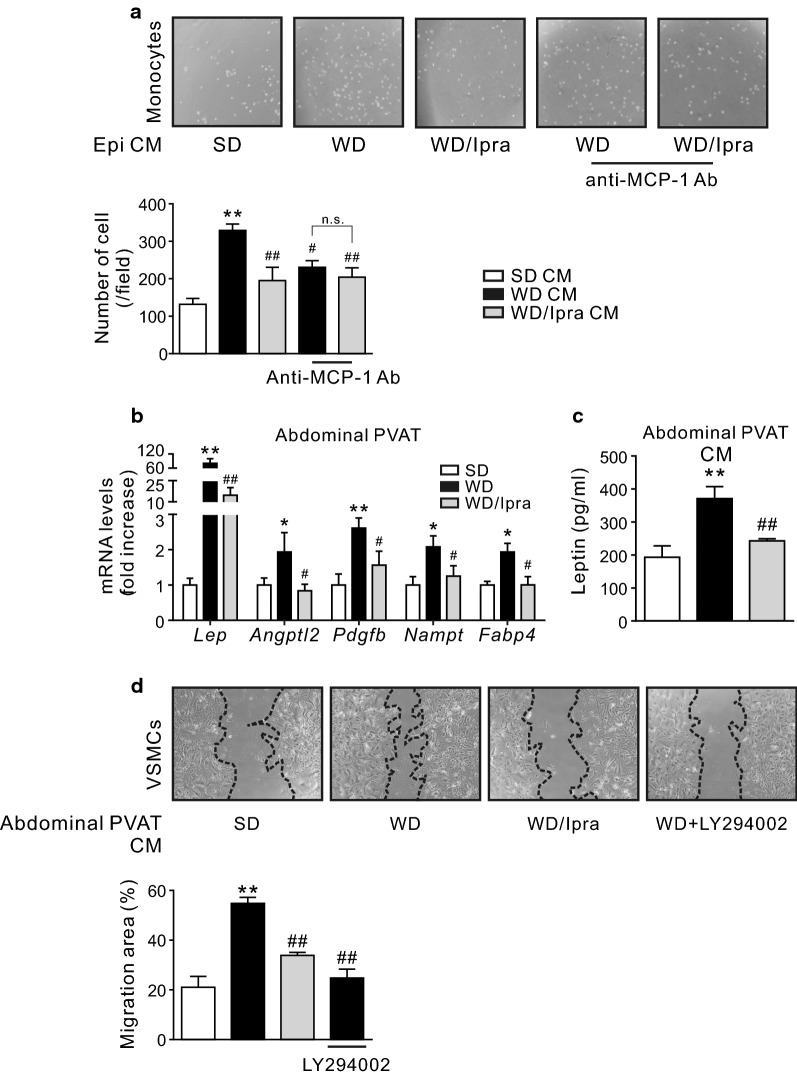
Ipra suppresses monocytes and VSMCs migration. **a** Representative pictures and quantitative data of migrated monocytes (WEHI274.1 cells) under CM of Epi with or without anti-MCP-1 blocking antibody (anti-MCP-1 Ab, 2 μg/ml). **b** Expression levels of proliferation- and migration-related genes in abdominal PVAT. **c** Leptin concentration in CM of abdominal PVAT. **d** Representative pictures and quantitative data of PDGF-BB-induced primary cultured VSMCs migration under CM of abdominal PVAT with or without LY294002 (10 μM). VSMCs, vascular smooth muscle cells. *ns* not significant. *p < 0.05, **p < 0.01 vs SD. ^#^*p* < 0.05, ^##^*p* < 0.01 vs WD. n = 6–8

We further examined whether changes of secretory factors from adipose tissue of Ipra-treated mice affect functions of macrophages and VSMCs related to atherosclerosis and vascular remodeling. An in vitro chemotaxis assay revealed that CM of Epi from vehicle-treated mice fed a WD significantly enhanced monocyte migration as compared to that from SD-fed mice, whose effect was attenuated in CM of Epi from Ipra-treated mice (Fig. 3a). Pretreatment with a neutralizing anti-MCP-1 antibody also inhibited the increase of monocyte migration by CM of Epi from vehicle-treated mice, and it also diminished the difference of monocyte migration stimulated by CM of Epi from Ipra- and vehicle-treated mice (Fig. 3a).

Leptin enhances VSMCs proliferation, migration, and neointimal hyperplasia via a PI3 K-dependent fashion [25]. In abdominal PVAT of Ipra-treated mice, including *Lep*, expression of *Angptl2* [26], *Pdgfb* [27], and *Nampt* [28], whose products have been shown to promote VSMCs proliferation, migration, and/or neointimal hyperplasia, were significantly suppressed compared to that of vehicle-treated mice (Fig. 3b). Leptin concentration in CM of abdominal PVAT from Ipra-treated mice was also significantly lower than that from vehicle-treated mice (Fig. 3c). Whereas the CM of abdominal PVAT from vehicle-treated mice enhanced platelet-derived growth factor (PDGF)-BB-induced VSMCs migration in vitro, its effect was significantly attenuated in CM of abdominal PVAT from Ipra-treated mice (Fig. 3d). Furthermore, pretreatment with LY294002, a PI3K inhibitor, also inhibited VSMCs migration by the CM of abdominal PVAT from vehicle-treated mice (Fig. 3d). Concentration of fatty acid binding protein 4 (FABP4), which is also reported to enhance VSMCs proliferation and migration [29], and inflammatory responses in macrophages [30], was significantly lower in CM of Epi from Ipra-treated mice, and showed trend of decrease in CM of abdominal PVAT from Ipra-treated mice (Additional file 1: Fig. S2a). Plasma concentration of FABP4 was lower in Ipra-treated mice compared to that of vehicle-treated mice (Additional file 1: Fig. S2b).

### Perivascular implantation of adipose tissue from Ipra-treated mice suppresses cuff-induced neointimal hyperplasia and vascular remodeling in ApoE-knockout mice

**Fig. 4:**
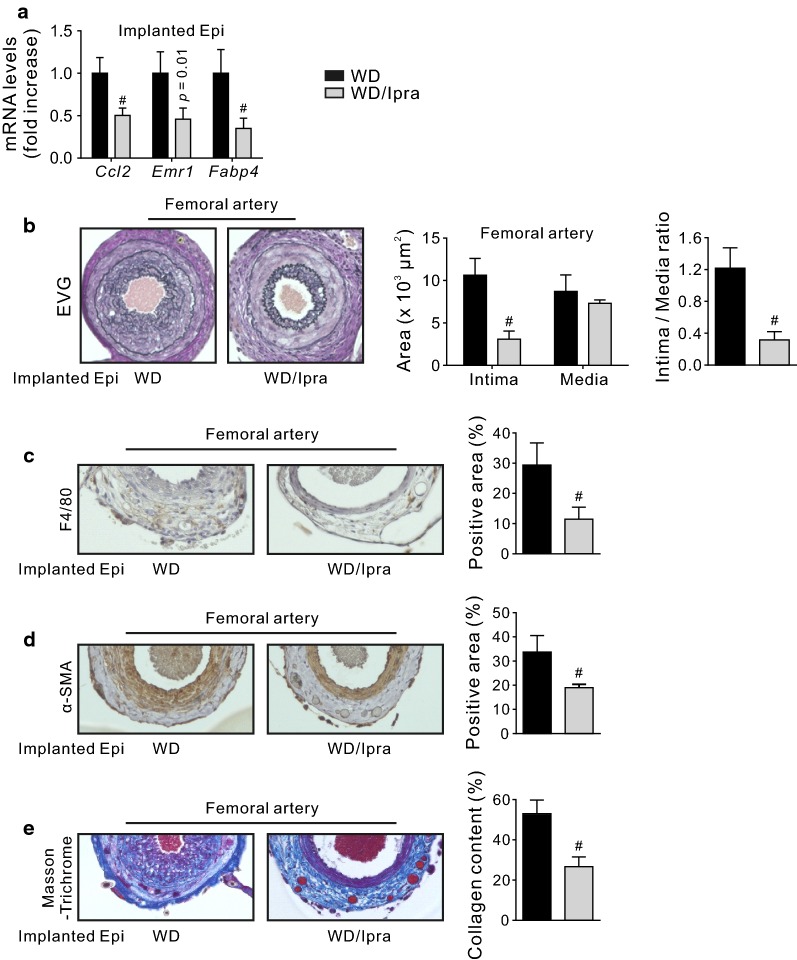
Perivascular implantation of adipose tissue from Ipra-treated mice suppresses cuff-induced neointimal hyperplasia and vascular remodeling in ApoE-knockout mice. **a** Gene expression of *Ccl2*, *Emr1* and *Fabp4* in implanted Epi after 4 weeks of implantation. **b** Representative pictures of EVG staining, and quantitative data of intima and media area in femoral arteries of ApoE-knockout mice 4 weeks after surgery. **c** F4/80 and **d** α-SMA immunostaining, and **e** Masson-Trichrome staining. *EVG* Elastica-van Gieson, *SMA* smooth muscle actin. Original magnification, ×200. ^#^*p* < 0.05 vs WD. n = 7

We finally asked whether the changes of characters in adipose tissue by Ipra affects neointimal hyperplasia and vascular remodeling in vivo, using the femoral artery cuff model; Epi from vehicle- or Ipra-treated mice fed a WD were implanted over a cuff placed around a femoral artery of ApoE-knockout mice. Four weeks after surgery, viable implanted Epi with vascularization were confirmed (Additional file 1: Fig. S3). Downregulation of *Ccl2*, *Emr1*, and *Fabp4* in implanted Epi from Ipra-treated mice remained 4 weeks after surgery (Fig. 4a). Neointimal hyperplasia assessed by intima area and intima to media ratio were significantly attenuated in ApoE-knockout mice implanted with Epi from Ipra-treated mice compared to vehicle-treated mice (Fig. 4b). The reduced neointimal hyperplasia accompanied with suppressed macrophage infiltration, VSMCs proliferation, and fibrosis (Fig. 4c–e).

## Discussion

The present study demonstrated that Ipra increased adipocyte size in abdominal PVAT in WD-induced obese and diabetic mice, which is consistent with our previous observation in Epi of diet-induced obese mice treated with SGLT2 inhibitors [19, 31, 32]. The adipocyte hypertrophy in abdominal PVAT accompanied a decrease of inflammation and fibrosis, which corresponded to “healthy adipose expansion” [33]. In addition to multiple metabolic benefits by SGLT2 inhibitors such as improvement of hyperglycemia, obesity, and dyslipidemia that could influence cardiovascular mortality, the present study proposed novel mechanisms by which SGLT2 inhibitors prevented vascular complications in T2DM via modulating PVAT characters.

### Possible mechanisms of the “healthy adipose expansion” in abdominal PVAT

We previously observed same phenotypes of the adipose expansion in Epi of Ipra-treated HFD-fed mice [13]; increased adipocyte size and reduced number of CLS were observed in Epi of Ipra-treated mice fed a HFD. It suggests that Ipra increased the lipid-storage capacity of adipocytes and inhibited adipocyte death followed by macrophage accumulation. These results are consistent with a study using adipocyte-specific inducible phosphatase and tensin homologue (PTEN)-knockout mice, which exhibit enhanced insulin signaling in adipocytes. Whereas PTEN-knockout mice gained more adipose tissue during HFD feeding, they showed enhanced insulin sensitivity, improved hepatic steatosis, and reduced adipose tissue inflammation [34]. Then, improvement of insulin resistance in adipocytes could cause the adipose expansion without deteriorating inflammation, and might lead to attenuation of ectopic lipid accumulation, which can be called as “healthy adipose tissue expansion”.

As a common action of PTEN-knockout mice, Ipra treatment improved insulin resistance via its insulin-independent glucose-lowering action; indeed, the present study has shown that Ipra treatment improves both hyperglycemia and hyperinsulinemia with increased Akt phosphorylation in abdominal PVAT compared to vehicle treatment. Although phosphorylation of Akt is not a specific marker of insulin signaling, improved hyperglycemia, hyperinsulinemia, and systemic insulin resistance assessed by HOMA-R indirectly suggest that increased phosphorylation of Akt is presumed to reflect improved insulin sensitivity in abdominal PVAT of Ipra-treated mice. Furthermore, Ipra treatment significantly increased Epi weight to body weight ratio despite of comparable whole body weight to vehicle-treated mice, suggesting the “healthy adipose tissue expansion”. Taken together, these observation suggest that enhanced insulin signaling in abdominal PVAT by Ipra was likely to play an main role in promoting the “healthy adipose expansion”. The detailed molecular mechanisms by which enhancement of insulin signaling in adipocytes causes the “healthy adipose expansion” require further studies.

### Cell death in PVAT and vascular injury

In addition to reduced expression of inflammation-related genes, increased adipocyte size, and reduced number of CLS and TUNEL-positive cells were observed in abdominal PVAT of Ipra-treated mice. It suggests that Ipra increases the lipid-storage capacity of adipocytes and inhibited adipocyte death followed by macrophage accumulation as we previously reported in Epi [19, 32]. Indeed, expression of HMGB1 protein, which is passively released by injured or dying cells and aggravates inflammatory processes in various cells [35], was markedly reduced in abdominal PVAT of Ipra-treated mice compared to that of vehicle-treated mice. HMGB1 is known as not only a hallmark of cell death, but also a cytokine; in blood vessels, high levels of extracellular HMGB1 have been detected in human atherosclerotic plaque [36], and are reportedly implicated in vascular inflammation by potentiating inflammatory responses including macrophage migration and activation [23, 24]. In VSMCs, it has been shown that HMGB1 modulates their phenotype toward the activated synthetic phenotype and stimulates MCP-1 gene expression through toll-like receptor 4 [24, 37]. Taken together, as well as the altered gene expression in abdominal PVAT, decreased adipocyte death in abdominal PVAT by Ipra is also considered to inhibit the development of vascular dysfunction and remodeling, especially in the setting of obesity as in the present study.

### Possible mechanisms of suppressed cuff-induced vascular remodeling

In the present study, perivascular implantation of Epi from Ipra-treated mice prevented cuff-induced neointimal hyperplasia and vascular remodeling in femoral arteries of ApoE-knockout mice compared to that from vehicle-treated mice. It suggests that Ipra-induced changes of secretory factors from Epi of WD-fed mice may attenuate these histological changes in the arteries. We implanted Epi instead of PVAT due to technical difficulties, however, it is supposed that PVAT-derived secretory factors would also affect cuff-induced vascular remodeling as paracrine manner. Although dissecting the responsible secretory factors changed by Ipra is beyond the present study, we assumed that, considered from ex vivo and in vitro studies, decreased HMGB1, leptin, MCP-1, NAMPT, PDGF-B and/or FABP4 expression/production from implanted adipose tissue could play inhibitory roles for the development of neointimal hyperplasia and vascular remodeling via attenuating monocyte migration and VSMCs proliferation. HMGB1 and MCP-1 reportedly play roles mainly in inflammatory cells such as macrophages, neutrophils, and lymphocytes to recruit them into the vascular adventitia. It has been shown that neutralization of HMGB1 reduces development of atherosclerosis in apolipoprotein E-deficient mice [38], suggesting significant contribution of decreased HMGB1 by Ipra to the inhibition of vascular remodeling. In contrast, leptin, PDGF-B, and visfatin, although they are also reported to affect inflammatory cells [39, 40], can affect VSMCs to enhance phenotype switch to proliferative character, and promote migration to the neointima [6, 39, 41]. In addition, FABP4 has been reported to induce proliferation and migration of vascular smooth muscle cells through a MAPK-dependent pathway [29]. In macrophages, treatment with recombinant FABP4 significantly increased gene expression of inflammatory markers in a dose-dependent manner [42]. FABP4 is also reported to ectopically express in endothelial cells to promote neointima formation by wire-induced vascular injury [43]. Then, decreased gene and/or protein expression of these factors derived from abdominal PVAT are presumed to coordinately and complementarily affect vascular cells, leading to inhibition of cuff-induced vascular remodeling.

### Persistent of SGLT2 inhibitors-induced changes in implanted adipose tissues

Moreover, it is noteworthy that expression pattern of pro-inflammatory genes in implanted adipose tissues partly remained similar to that before implantation even 4 weeks after implantation. It suggests some cell-autonomous mechanisms that maintain altered gene expression, referred to as “cellular memory”, after exposure to SGLT2 inhibitors, or SGLT2 inhibitors-induced changes of extracellular factors. As one of the possible mechanism to maintain the “cellular memory”, epigenetic modifications are generally speculated; obesity and/or diabetes are shown to associate DNA and histone methylation levels in various genes of adipose tissue [44], including inflammatory genes [45, 46]. Whereas evidences related to SGLT2i-induced epigenetic changes are limited, a previous paper has shown that treatment with SGLT2i affects gene expression in aortae associated with epigenetic modifications; treatment with empagliflozin for 6 week prevented induction of inflammation- and glucotoxicity-related genes in aortae of Zucker fatty rats, along to suppression of activating epigenetic mark trimethylation of histone H3 at lysine4 (H3K4me3) of these genes [47]. It is therefore possible that Ipra attenuates WD-induced changes of inflammatory genes associated with epigenetic changes in adipose tissue, and the epigenetic changes may contribute to maintain the gene expression levels even after Epi extraction. The precise molecular, genetic, and epigenetic mechanisms by which gene expression are maintained in adipose tissue from Ipra-treated mice needs to be elucidated.

### Differences between thoracic and abdominal PVAT

Unlike abdominal PVAT, Ipra did not affect adipocyte size, and *Ccl2, Ccr2*, or *Emr1* expression in thoracic PVAT. It has been known that the structural and physiological characteristics of PVAT vary according to its location. Abdominal PVAT resembles white adipose tissue (WAT), with less differentiated adipocytes, poor vascularization, a specific profile of cytokines production, and contains infiltrates of macrophages [48]. On the other hand, thoracic PVAT exhibits features that resemble brown adipose tissue (BAT) rather than WAT [49, 50]. Although precise mechanisms by which Ipra differently affects thoracic and abdominal PVAT remains unclear, the distinct differences between thoracic and abdominal PVAT in response to Ipra may attribute these basic cellular characteristics. Furthermore, a previous report has suggested that insulin-induced Akt phosphorylation is more pronounced in WAT than BAT in mice [51], suggesting that WAT appears to be more insulin-sensitive than BAT. Assuming that insulin sensitivity is crucial for promoting the healthy adipose expansion, the difference of insulin sensitivity may result in the differences of characters between thoracic and abdominal PVAT.

## Conclusions

The present study proposes a novel mechanism by which a SGLT2 inhibitor Ipra induced adipocyte hypertrophy without increasing inflammation, fibrosis, and adipocyte death in abdominal PVAT of WD-fed mice. The data of this study also imply a novel inter-organ network between kidney, adipose tissue and vasculature, and thus suggest a clinical implication for the prevention and treatment of obesity and/or T2DM-associated cardiovascular complications through pharmacological intervention.

## Additional file

**Additional file 1.** Additional figures and tables.

## Data Availability

The datasets used and/or analyzed during the current study are available from the corresponding author on reasonable request.

## Abbreviations

PVAT: perivascular adipose tissue
Ipra: ipragliflozin
HMGB1: high mobility group box 1
CM: conditioned media
SGLT2: sodium-glucose cotransporter 2 inhibitor
T2DM: type 2 diabetes mellitus
WAT: white adipose tissue
BAT: brown adipose tissue
MCP-1: monocyte chemoattractant protein-1
FABP-4: fatty acid binding protein 4
VSMCs: vascular smooth muscle cells
