# Supplementary material for: Ipragliflozin-induced adipose expansion inhibits cuff-induced vascular remodeling in mice
Source: Cardiovasc Diabetol. 2019 Jun 24;18:83. doi: 10.1186/s12933-019-0886-1 (PMC6589884; doi:10.1186/s12933-019-0886-1)

SUPPLEMENTAL MATERIAL

Ipragliflozin-induced adipose expansion inhibits cuff-induced vascular remodeling in mice

Kentaro Mori, Kyoichiro Tsuchiya, Suguru Nakamura, Yasutaka Miyachi, Kumiko Shiba, Yoshihiro Ogawa, Kenichiro Kitamura

SUPPLEMENTAL FIGURE LEGENDS

Supplemental Figure 1. Plasma lipid profile, and histology and gene expression in thoracic PVAT of Ipra-treated WD-fed mice.

(A) Epi weight to body weight ratio of Ipra-treated WD-fed mice. (B) Plasma total cholesterol, triglyceride and non-esterified fatty-acid (NEFA) levels after 10 weeks of Ipra treatment. (C) Hematoxylin and eosin (HE) staining, and (D) gene expression levels in thoracic PVAT 10 weeks after Ipra treatment. NEFA, Non-esterified Fatty Acid. Original magnification, × 200. * $p < 0.05$, ** $p < 0.01$ vs SD. n = 6-8.

Supplemental Figure 2. Concentration of FABP4 in conditioned medium of Epi and plasma of Ipra-treated WD-fed mice.

Concentration of FABP4 in (A) conditioned medium (CM) of Epi and abdominal PVAT, and (B) plasma of SD or Ipra-treated WD-fed mice. ** $p < 0.01$ vs SD, # $p < 0.05$, ## $p < 0.01$ vs WD. n = 6-8.

Supplemental Figure 3. Implanted adipose tissue 4 weeks after surgery.

A representative picture of implanted Epi 4 weeks after surgery.

Supplemental Table. List of primers.

Fabp4	Fw	AGACGACAGGAAGGTGAAGA
	Rv	TAACACATTCCACCACCAGC
Nampt	Fw	CAGTGGCCACAAATTCCAGA
	Rv	CCTATGCCAGCAGTCTCTTG
Angptl2	Fw	CACCTACAACCGCATCATCA
	Rv	TCCATGGACCTGATGGCTTA
Pdgfb	Fw	CTTCCTCTCTGCTGCTACCT
	Rv	AGCCCCATCTTCATCTACGG
Ccl2	Fw	CCACTCACCTGCTGCTACTCAT
	Rv	TGGTGATCCTCTTGTAGCTCTCC
Emr1	Fw	CTTTGGCTATGGGCTTCCAGTC
	Rv	GCAAGGAGGACAGAGTTTATCGT
Ccr2	Fw	ACAAATCAAAGGAAATGGAAGAC
	Rv	TGCCGTGGATGAACTGAGG
Col1a1	Fw	CCTCAGGGTATTGCTGGACAAC
	Rv	ACCACTTGATCCAGAAGGACCTT
Col1a2	Fw	TTGCAATCGGGATCAGTACGA

	Rv	CACGTGGTCCTCTGTCTCCAG
Fn1	Fw	GCTGAAAGGACCCAATGTCT
	Rv	AGAATCCATCCCCACAGGAA
Lep	Fw	TCCAGGATGACACCAAAACCC
	Rv	TGAAGTCCAAGCCAGTGACC
36b4	Fw	GGCCCTGCACTCTCGCTTTC
	Rv	TGCCAGGACGCGCTTGT

Fw: forward primer, Rv: reverse primer

Supplemental Figure 1

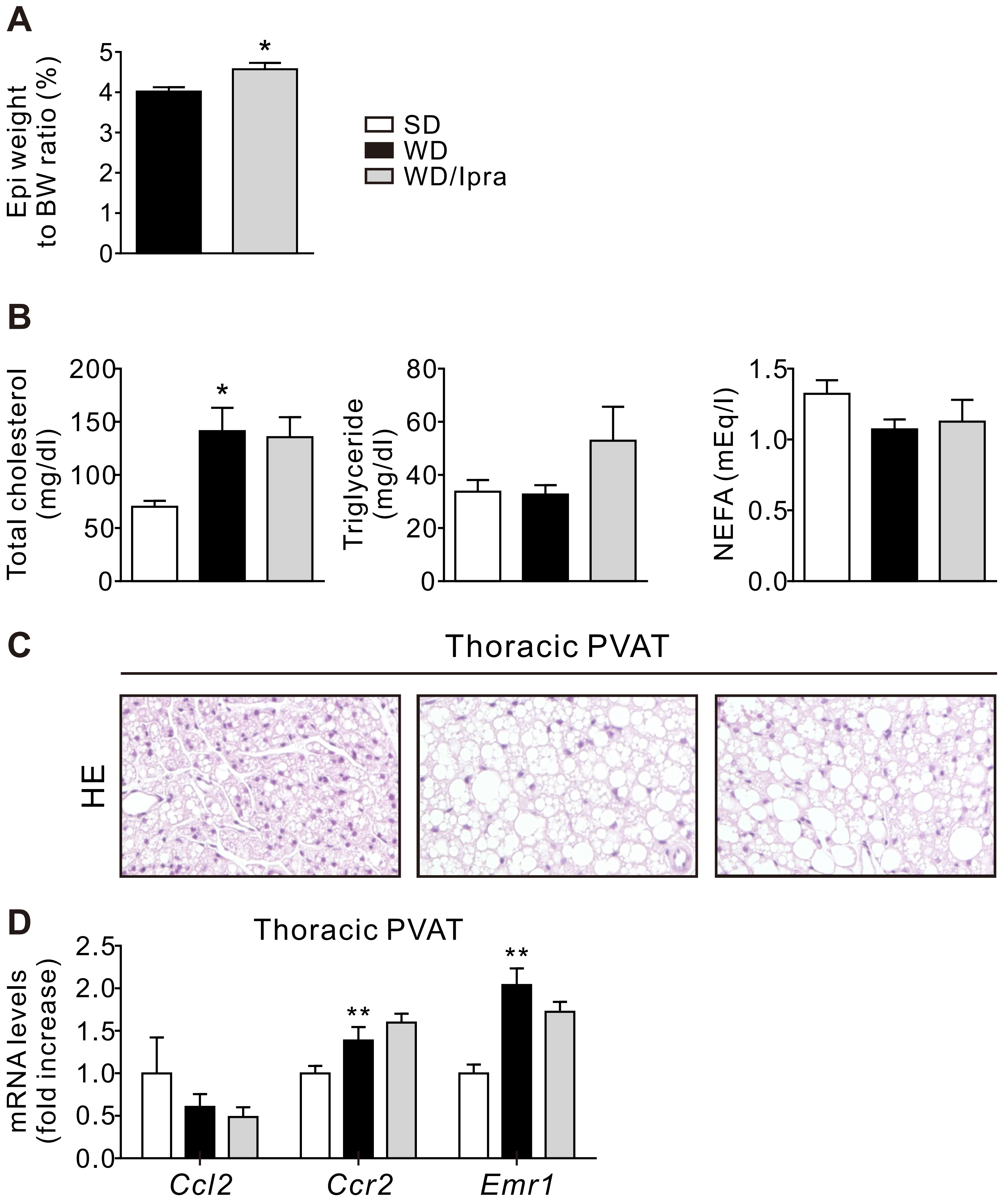

Supplemental Figure 2

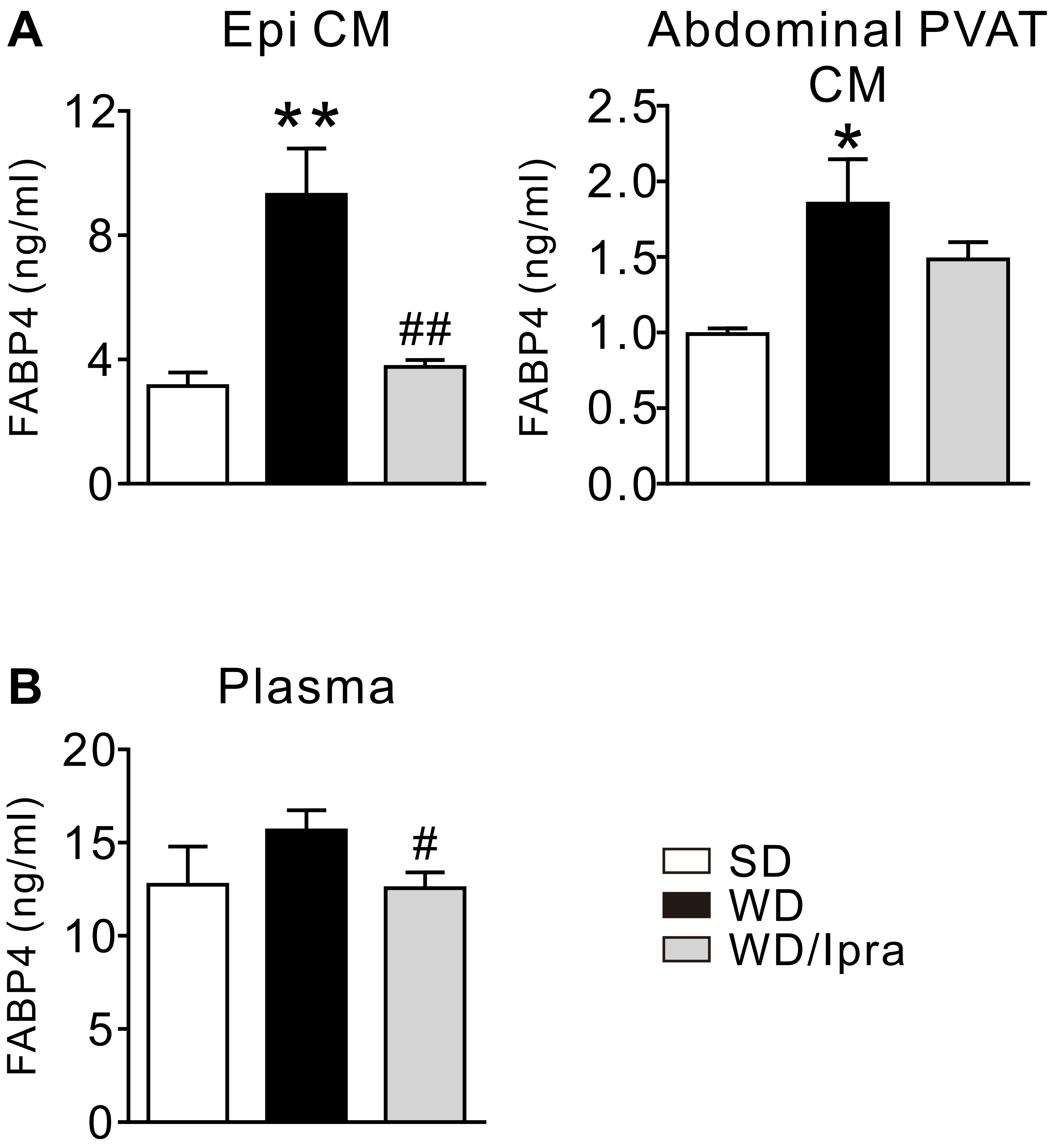

Supplemental Figure 3

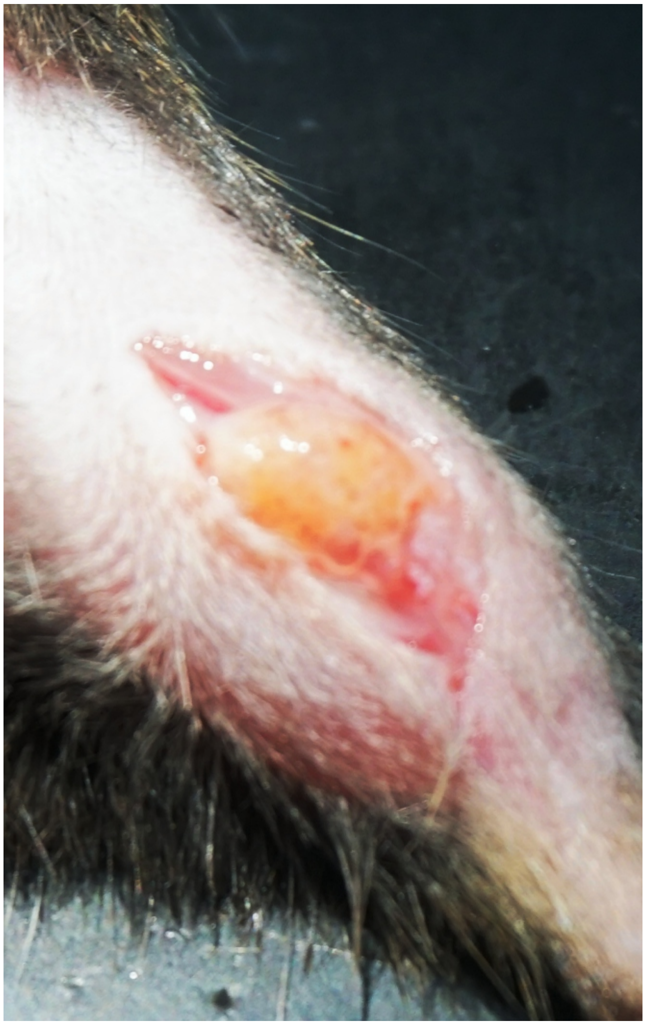

Supplement: Supplementary file 1 — Additional file 1. Additional figures and tables. [file 12933_2019_886_MOESM1_ESM.pdf]
